# Alpha-fetoprotein activates AKT/mTOR signaling to promote CXCR4 expression and migration of hepatoma cells

**DOI:** 10.18632/oncoscience.115

**Published:** 2015-01-06

**Authors:** Mingyue Zhu, Junli Guo, Hua Xia, Wei Li, Yan Lu, Xu Dong, Yi Chen, Xieju Xie, Shigan Fu, Mengsen Li

**Affiliations:** ^1^ Hainan Provincial Key Laboratory of Carcinogenesis and Intervention, Hainan Medical College, Haikou, PR.China; ^2^ Key Laboratory of Molecular Biology, Hainan Medical College, Haikou, PR.China; ^3^ Graduate School, Guanxi Medical University, Nanning, PR. China; ^4^ Department of Pathophysiology, Hainan Medical College, Haikou, PR.China; ^5^ Department of Physiology, Hainan Medical College, Haikou, PR.China; ^6^ Institution of Tumor, Hainan Medical College, Haikou, PR.China

**Keywords:** Alpha fetoprotein, AKT/mTOR signal, CXCR4, Hepatocellular carcinoma, Metastasis

## Abstract

CXCR4, stromal cell-derived factor-1α(SDF 1α) receptor, stimulates growth and metastasis of hepatocellular carcinoma (HCC). Alpha-fetoprotein(AFP) governs the expression of some metastasis-related genes. Here we report that AFP and CXCR4 levels correlated in HCC tissues. AFP-expressing vectors induced CXCR4. In agreement, AFP depletion by siRNA decreased CXCR4. AFP co-localized and interacted with PTEN, thus inducing CXCR4 by activating AKT(Ser473) phosphorylation. In turn, phospho-mTOR(Ser2448) entered the nucleus and bound the CXCR4 gene promoter. Thus, AFP promoted migration of HCC cells. In concusion, AFP induced CXCR4 by activating the AKT/mTOR signal pathway.

## INTRODUCTION

Hepatocellular carcinoma(HCC), a highly aggressive and deadly malignancy representing the most common cancers worldwide, is particularly prevalent in Eastern Asia including China, Taiwan and Korea[1,2]. Many patients with early disease are asymptomatic; Therefore, the prognosis is extremely unsatisfactory with high incidences of postoperative local recurrence or distant metastasis that lead to poor survival rates of patients with HCC[3].

Alpha-fetoprotein (AFP), a tumor-associated fetal protein, has been employed as a useful marker for HCC detection and monitoring[4], AFP and has been purified, characterized, cloned, and sequenced for use in the clinical diagnostic laboratories[5]. In recent years, our laboratory has reported that AFP functions as an intracellular signaling molecule of great significance in the development and growth of malignant liver tumor cells. Our studies have demonstrated that AFP has the capability a capacity to contribute to the proliferation of both tumor and normal cells[6] and facilitate the liver cancer cells escaping from immune surveillance by blocking the caspase signaling pathway of tumor cells, and triggering the Fas/FasL interaction between tumor cells and lymphocytes[7]. We also reported that AFP was able to block RA-RAR signaling to promote tumor cell growth[8] and interrupt the onward transduction of apoptotic signaling by binding with caspase-3[9]. Moreover, subsequent studies demonstrated that cytoplasmic AFP was able to activate PI3K/AKT pathway by causing dysfunction of PTEN protein dysfunction, leading to aberrant proliferation of HCC cells growth[10]. Evidence now supports the possibility that intracellular AFP functions as a signal regulator related to apoptosis signaling and affects HCC growth. However, it is unknown whether the biological role of AFP is involved in regulating the metastasis factors of tumor progression, which is yet another important and interesting aspect of AFP since serum AFP level was significantly correlated with tumor progression and patient survival time[11,12].

CXC motif chemokine receptor 4(CXCR4) is a CXC chemokine receptor that was initially found to be essential for the entry of HIV-1 into host cells[13]. To date, CXCR4 has been demonstrated to be involved in various aspects of HCC initiation and progression, specifically migration, invasion, and metastasis[14,15]. Xiang et al[16] reported that CXCR4 expression was associated with and predictive of bone metastasis in patients with HCC and decreased overall median survival. A subsequent study showed that the nuclear accumulation of CXCR4 was is positive correlated with a higher risk of lymph node metastasis in HCC and a poor outcome[17]. CXCR4 expression is regulated by activated PI3K/AKT signaling in HCC[18], and phospho-mTOR(p-mTOR) is a downstream molecule of the PI3K/AKT signal that enters the nucleolus to regulate target gene expression, including CXCR4. These findings showed that p-mTOR plays a role in HCC metastasis.

Therefore, the purpose of this study is to investigate the role of AFP in on regulating expression of CXCR4, a critical regulator of metastasis, and the underlying mechanisms in clinical HCC tissues as well as in the PLC/PRF/5(AFP-producing) and HLE(non-AFP-producing) HCC cell lines. Although AFP has been well known as the “gold standard” among tumor-specific molecular biomarkers since the 1970s[19], whether intracellular AFP serves as a regulator associated with modulating HCC invasion and metastasis still unclear has not been investigated until now. The present findings will indicate the intrinsic properties of AFP to affect the signaling pathway involved in the regulation of metastasis factor CXCR4 and provide a new perspective on the biological role of AFP.

## RESULTS

### AFP positive associated with CXCR4 expression and interacted with PTEN in clinical HCC patients' tissues

**Figure 1:**
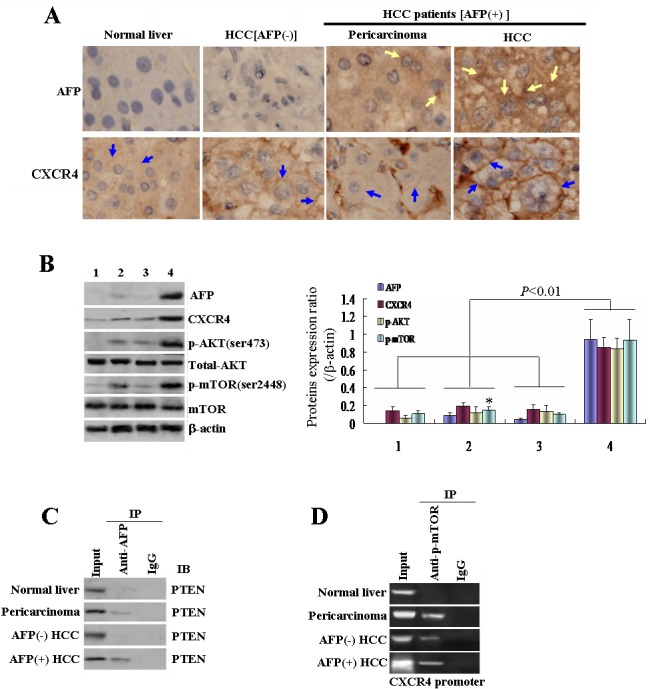
Effects of alpha-fetoprotein(AFP) on expression of CXCR4 in hepatocellular carcinoma(HCC) clinical patients' sample tissues A, AFP and CXCR4 expressions in normal liver tissues, AFP-negative HCC tissues, and AFP-positive HCC tissues were detected using immunohistochemical staining(IHC). B, Expressions of AFP, CXCR4, p-AKT(Ser473), and p-mTOR(Ser2448) were detected by Western blotting: 1, normal liver tissue; 2, AFP(−) HCC tissue; 3 pericarcinoma tissue; 4, AFP(+) HCC tissue; right column graph indicated the quantity of protein expression, *P<0.05 vs normal liver tissue group and pericarcinoma tissue group. C, The interaction combination of AFP with and phosphatase and tensin homolog deleted on chromosome 10(PTEN) in the liver tissues was analyzed by co-immunoprecipitation. D, p-mTOR(Ser2448) binds interactions with the CXCR4 gene promoter as detected by chromatin immunoprecipitation. The yellow arrows indicate AFP location and expression in the liver tissues, while the blue arrows indicate CXCR4 location and expression in the liver tissues. We performed three independent experiments in triplicate.

Firstly, we investigated AFP and CXCR4 expression in the tissues of normal liver, HCC with AFP(−), and HCC with AFP(+) and its pericarcinoma. As shown in Figure 1A, the immunohistochemical(IHC) results revealed that the specific stronger staining of AFP and CXCR4 appeared in the HCC patients with AFP(+) tissues compared with its pericarcinoma, HCC patients with AFP(−), and normal liver tissues. The levels of AFP, CXCR4, phospho-AKT(Ser473)[p-AKT(Ser473)], and phospho-mTOR(Ser288 4) [p-mTOR(Ser2448)] proteins in the specimens from the tissues of normal liver, HCC patients with AFP(−), and HCC patients with AFP(+) and its pericarcinoma were evaluated by Western blotting. Consistent with the IHC results, high AFP and CXCR4 expressions were emerged seen in the tumor HCC tissues with AFP(+) compared to the adjacent non-cancerous liver tissues, HCC patients with AFP(−), and normal liver tissues(Figure1B), implicated suggesting that CXCR4 overexpression is closely correlated to HCC patients with AFP(+). It was noted that the p-AKT(Ser473) and p-mTOR(Ser2448) protein levels were also significantly markedly elevated in the cancerous HCC patients with AFP(+) tissues (Figures1B), demonstrateding that AKT/mTOR signaling pathway activation was involved in pathological progressing pathogenesis of HCC patients with AFP(+). Co-IP analysis results was used to further confirmed the interaction between AFP and PTEN in the clinical specimens as observed in the cancerous HCC patients with AFP(+) tissues, and immunoprecipitation of the putative AFP-PTEN protein complex with anti-AFP antibody led to the obvious detection of an AFP band (Figure 1C), the finding that was consistent with our previous report in hematoma cell lines[10]. When p-mTOR(Ser2448) levels were increased as shown by the ChIP assay (Figure 1D), the binding of p-mTOR(Ser2448) to the promoter region of the *CXCR4* gene was also elevated, indicating the role of p-mTOR(Ser2448) in regulating expression of *CXCR4* expression.

### AFP promoted expression of CXCR4 in HCC cell lines

**Figure 2:**
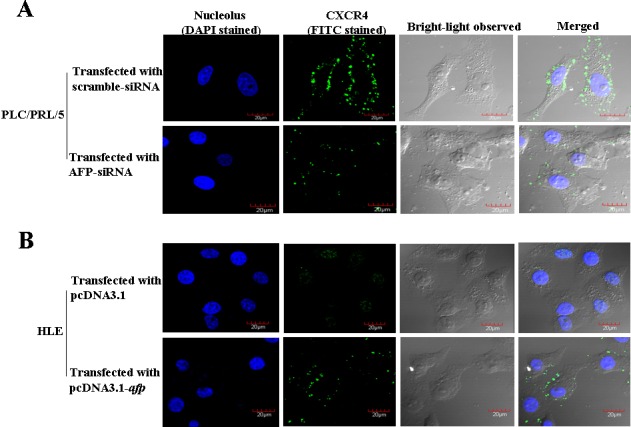
Influence of AFP on the expression of CXCR4 in PLC/PRF/5 cells and HLE cells observed by laser confocal microscopy A, Human liver cancer PLC/PRF/5(AFP-producing) cell line was transfected with AFP-siRNA vectors for 48 hours and CXCR4 expression was observed by laser confocal microscopy. B, human liver HLE(non-AFP-producing) cell lines were transfected with pcDNA3.1-*afp* vector for 48 hours and CXCR4 expression was observed by laser confocal microscopy. The images were taken of three independent experiments.

PLC/PRF/5 cells (AFP-producinger) and HLE cells (non AFP-producinger) were used to further examine the effect of AFP on CXCR4 expression in human liver cancer cell lines in the present study. As shown by laser confocal laser microscopy in Figure 2, treatment of highly AFP-expressed PLC/PRF/5 cells with AFP-siRNAs led to a significant decrease in CXCR4 expression(Figure 2A), however, expression of CXCR4 significantly stimulated while HLE cells were transfected with pcDNA3.1-*afp* vectors, and CXCR4 located membrane of the cells(Figure 2B). These This results proved that AFP stimulated CXCR4 expression and location in celluar membrane.

### AFP induced phosphorylation of AKT(Ser473) related to promote expression of CXCR4 in HCC cell lines

**Figure 3:**
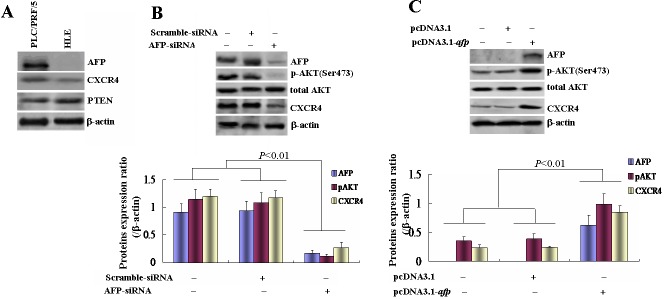
Effects of AFP on p-AKT(Ser473) and CXCR4 expressions of PLC/PRF/5 and HLE cells A, Expressions of AFP, CXCR4, and PTEN in PLC/PRF/5 or HLE cells were analyzed by Western blotting. B, PLC/PRF/5 cells were transfected with AFP-siRNA vectors for 48 hours and the expressions of AFP, p-AKT(Ser473), and CXCR4 were analyzed by Western blotting, low column graph indicated the quantity of protein expression. C, HLE cells were transfected with pcDNA3.1-*afp* vectors for 48 hours and the expressions of AFP, p-AKT(Ser473), and CXCR4 in HLE cells were analyzed by Western blotting, low column graph indicated the quantity of protein expression. We performed three separate experiments in triplicate.

In order to estimate expression of AFP, CXCR4 and PTEN in selected HCC cell lines, PLC/PRF/5 and HLE cells. Further, Western blotting analysis was used to detect the expression of these proteins. The results indicated that highly expression of AFP in PLC/PRF/5 cells but not in HLE cells(Figure 3A). Whereas, expression of PTEN and CXCR4 all emerged in PLC/PRF/5 and HLE cells(Figure 3A). When intracellular AFP was abolished by AFP-siRNAs in PLC/PRF/5 cells, the expressions of p-AKT(Ser473) and CXCR4 were significantly decreased contrast with non treated group and scramble-siRNA treated group(*P*<0.01)(Figure 3B). In addition, HLE cells(non AFP-producing) were transfected with pcDNA3.1*-afp* vectors, the expression of CXCR4 and p-AKT(Ser473) were significantly enhanced contrast with non treated group and pcDNA3.1 empty vector group(Figure 3C).

### Cytoplasmic AFP interacts with PTNE in HCC cell lines

**Figure 4:**
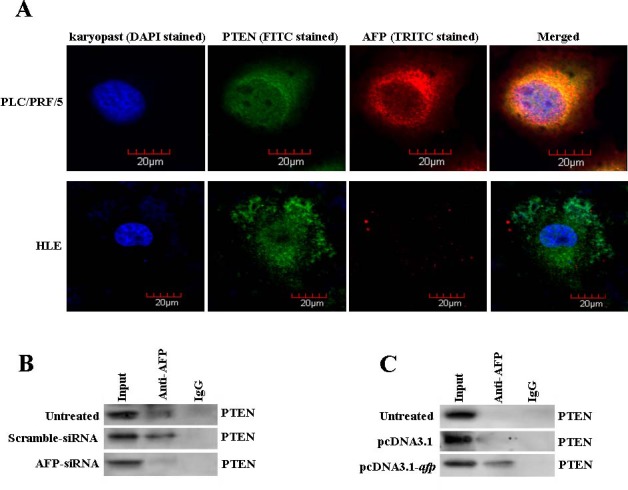
Co-location and interaction of AFP with PTEN in PLC/PRF/5 and HLE cells A, Co-location of AFP and PTEN in PLC/PRF/5 or HLE cells were observed by laser confocal microscopy. B, PLC/PRF/5 cells were transfected with AFP-siRNA vectors for 48 hours and the interaction of AFP with PTEN was detected by co-immunoprecipitation(Co-IP). C, HLE cells were transfected with pcDNA3.1-*afp* vectors for 48 hours and the interaction of AFP with PTEN was detected by Co-IP. We performed separate experiments in triplicate.

Consistent with our previous report[10], in this study, the laser confocal microscopy results showed that AFP co-localized with PTEN in the cytoplasm of AFP-producing PLC/PRF/5 cells but not in non AFP-producing HLE cells (Figure 4A). Co-IP analysis indicated that AFP was able to interaction with PTEN in PLC/PRF/5 cells, but the interaction vanished while the cells were transfecd with AFP-siRNA vectors(Figure 4B). AFP interaction with PTEN was undetectable in HLE cells, however, AFP combined with PTEN was emergence while HLE cells transfected with pcDNA3.1-*afp* vectors(Figure 4C). These results proved that AFP has a characteristic to interact with PTEN in HCC cells.

### AFP promotes expression of CXCR-4 and p-mTOR(Ser2448) through activating AKT signaling in HCC cell lines

**Figure 5:**
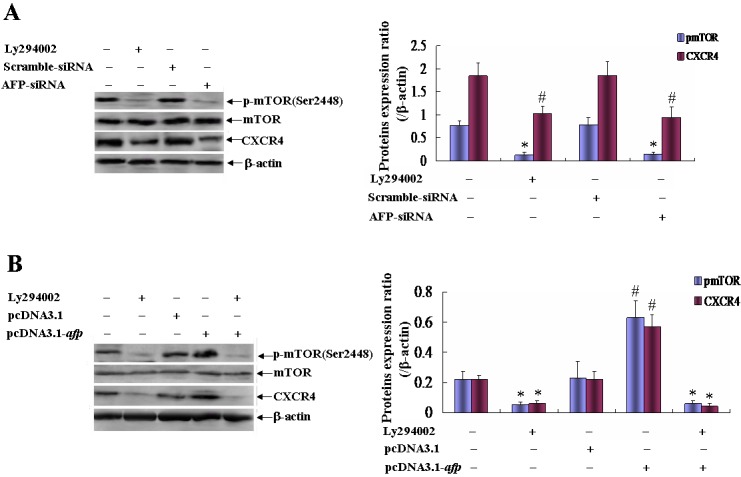
Effects of AFP on the expressions of p-mTOR(Ser2448) and CXCR4 in PLC/PRF/5 and HLE cells A, PLC/PRF/5 cells were treated with a potent inhibitor of PI3K/AKT, Ly294002 (20μmol/L), or transfected with AFP-siRNA for 48 hours, and the expressions of p-mTOR(Ser2448) and CXCR4 were analyzed by Western blotting, right column graph indicated the quantity of protein expression, *P<0.01 vs non-treated(control) group and scramble-siRNA vectors treated group; #*P*<0.01 vs control group and scramble-siRNA vectors treated group. B, HLE cells were treated with Ly294002 (20μmol/L) or pcDNA3.1-*afp* vectors or co-treated with Ly294002 (20μg/mL) and pcDNA3.1-*afp* for 48 hours, and the expressions of p-mTOR(Ser2448) and CXCR4 were analyzed by Western blotting, right column graph indicated the quantity of protein expression, *P<0.01 vs control group and pcDNA3.1 empty vectors treated group; #*P*<0.01 vs control group, Ly294002 treated group, pcDNA3.1 empty vectors treated group, and co-treated with Ly294002 (20 μg/mL) and pcDNA3.1-*afp* group. We performed separate experiments in triplicate.

We have demonstrated that AFP harbors a function to activate transduction of PI3K/AKT signal pathway[10]. To further analyze AFP regulated the expression of p-mTOR(Ser2448) and CXCR4 in HCC cells. Western blotting assay indicated that the expression of p-mTOR(Ser2448) and CXCR4 were significantly depressed while PLC/PRF/5 cells were transfected with AFP-siRNA vectors contrast with non-treated group and scramble-siRNA vectors treated group(*P*<0.01), and similar effects were occurred while PLC/PRF/5 cells were treated with Ly294002, a PI3K specific inhibitor(Figure 5A). Whereas, expression of p-mTOR(Ser2448) and CXCR4 were significantly elevated in HLE cells while transfected with pcDNA3.1-*afp* vectors contrast with non-treated group and pcDNA3.1 empty vectors treated groups(*P*<0.01), Ly294002 interrupted the role of pcDNA3.1-*afp* vectors in on promoting expression of p-mTOR(Ser2448) and CXCR4 in HLE cells(Figure 5B). The results suggested that AFP stimulated expression of CXCR4 involved in activating AKT/mTOR signaling pathway.

### AFP promotes p-mTOR(Ser2448) binding to *CXCR4* gene promoter

**Figure 6:**
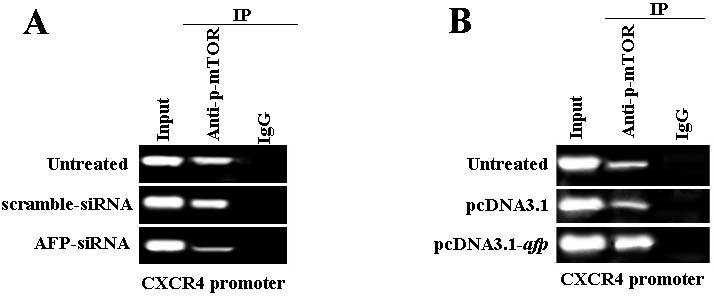
Effects of AFP on combination of p-mTOR(Ser2448) and the CXCR4 gene promoter in PLC/PRF/5 and HLE cells A, PLC/PRF/5 cells were transfected with AFP-siRNA for 48 hours, and the combination of p-mTOR(Ser2448) and the *CXCR4* gene promoter was evidenced by chromatin immunoprecipitation(ChIP). B, HLE cells were transfected with pcDNA3.1-*afp* vectors for 48 hours, and the combination of p-mTOR(Ser2448) with the CXCR4 gene promoter was evidenced by ChIP. We performed separate experiments in triplicate.

Reduced AFP in the AFP-siRNA transfected, ChIP results indicated that human hepatoma PLC/PRF/5 cells were transfected with AFP-siRNA vectors led to an apparent reduction of binding of p-mTOR(Ser2448) to the promoter elements in the 5′-flanking region of the *CXCR4* gene compared to the input(Figure 6A). For further confirmation, HLE cells were transfected with pcDNA3.1-*afp* vectors were able to promote greater binding of p-mTOR(Ser2448) to CXCR4 promoter element (Figure 6B). These ChIP assay results showed that AFP plays a key role in promoting p-mTOR(Ser2448) to upregulate *CXCR4* gene expression by binding to the response element in the regulatory region of *CXCR4*.

### AFP promotes migration metastasis of human hepatoma cells

**Figure 7:**
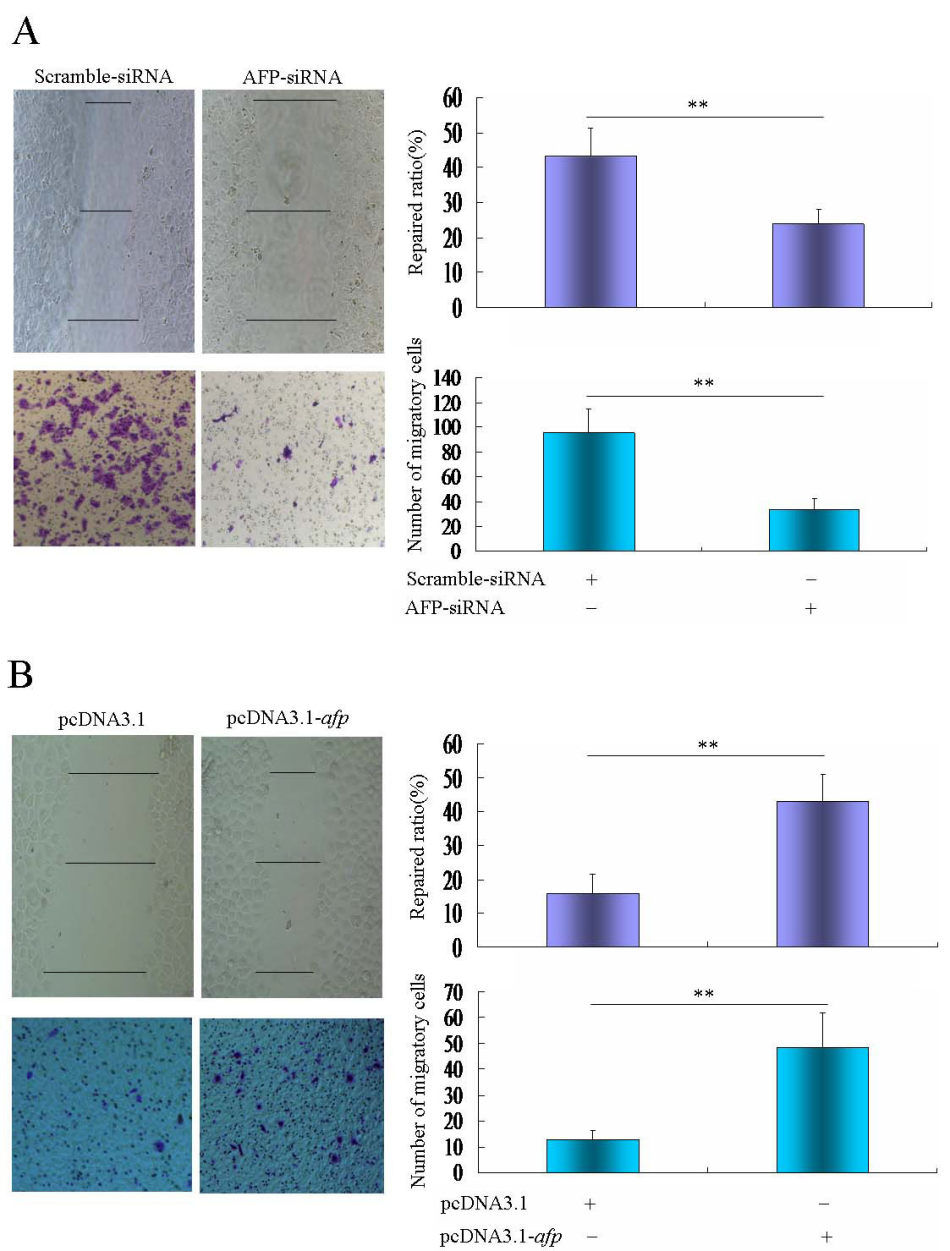
Effects of AFP knockdown or overexpression on wound healing and migration of PLC/PRF/5 and HLE cells A, PLC/PRF/5 cells were transfected with AFP-siRNA vectors for 48 hours, wound healing were observed by microscopy, and migratory cells were analyzed by a transwell chamber assay. B, HLE cells were transfected with pcDNA3.1-*afp* vectors for 48 hours, wound healing of the cells were observed by microscopy, and migratory cells were analyzed by a transwell chamber assay. **P<0.01. We performed three independent experiments.

We analyzed the effect of AFP on the movement ability of HCC cells using the scratch healing assay. The results indicated that while PLC/PRF/5 cells were transfected with AFP-siRNA vectors, the cells sealed the wound area were significantly degression after scratching contrast with scramble-siRNA vectors-transfected cells, which were significantly less efficient in healing as indicated by the mean wound repaired distances of 23.9±4.2% vs 43.4±8.3%(*P*<0.01). When HLE cells were transfected with AFP-expressing vectors(pcDNA3.1-*afp*), the cells sealed the wound area were significantly enhancement after scratching contrast with the pcDNA3.1 empty vectors-transfected cells that significantly increased healing efficient as indicated by the mean wound repair distances of 43.0±8.0 vs 15.7±6.0(*P*<0.01)(Figure 7A and 7B).

In present study, we employed cell migration assays to evaluate the role of AFP in metastasis of HCC cells. The results showed that repressed AFP expression by AFP-siRNA vectors in PLC/PRF/5 cells led to fewer migratory cells contrasted compared to scramble-siRNA vectors groups(34.0±8.5 vs 95.5±20.0; *P*<0.01); Nevertheless, when HLE cells were transfect with pcDNA3.1-*afp* vectors, migration of the cells were significantly increased were used to transfect into HLE cells, significantly more migratory HLE cells were appearance compared to pcDNA3.1 empty vectors groups (48.3±13.6 vs 12.5±3.7; *P*<0.01)(Figure 7A and 7B). These results revealed that AFP harbors a function to promote migration of HCC cells.

## DISCUSSION

The goal of the present study was to determine whether AFP can regulate the expression of CXCR4, a chemokine receptor that has been closely associated with tumor cell invasion and metastasis and the underlying mechanisms. In this study, the evidence from both clinical HCC tissues and HCC cells clearly indicated that AFP acts as a regulator to promote CXCR4 expression. Firstly, immunohistochemical staining and Western blotting analysis showed that CXCR4 was significantly upregulated in AFP-positive HCC patients' tissues compared with that in normal liver tissues, AFP-negative HCC patients' tissues, or adjacent noncarcinoma tissues. Secondly, this finding was further confirmed by the silencing of AFP by knockdown in AFP-producing PLC/PRF/5 cells or by transfection of the *afp* gene into non-AFP-producing HLE cells. Both clinical patients and cell culture showed that CXCR4 expression was positively correlated with AFP levels, which provided direct evidence of the regulatory effects of AFP in CXCR4 expression.

CXCR4 is a well-known pivotal regulator of tumor invasionveness and metastasis[21-25]. The results of multiple in vitro and in vivo preclinical models demonstrated that blocking function of CXCR4 receptor by a monoclonal antibody clearly restrained cancer cell proliferation, motility, and invasion[26,27]. Although it remains unclear how AFP leads to CXCR4 overexpression in HCC cells, previous reports implicated involvement of PI3K/AKT signaling pathway in regulating expression of CXCR4. Phillips et al.[28] indicated that epidermal growth factor and hypoxia promoted CXCR4 expression via PI3K/AKT/mTOR pathway in non-small cell lung cancer. Huang et al.[29] also reported that the PI3K/AKT pathway was involved in CXCR4 expression and the hepatocyte growth factor-induced activation of protein kinase C-ζ in human breast cancer cells. Moreover, Dubrovska et al.[30] demonstrated the direct regulation of CXCR4 expression by the PI3K pathway and thus implied a mutually positive regulatory feedback loop between the PI3K/AKT and CXCR4/CXCL12 signaling pathways, which are both important for cancer metastasis. Furthermore, our previous results indicated that HBV-X protein induced the expression of AFP in human normal liver cells, which correlated to CXCR4 expression in human hepatoma cells[31], and accumulated evidences showed that the mTOR/CXCR4 signal axis is closely associated with the promotion of cancer cell metastasis[32]. In the present study, we also explored the possible mechanisms by which AFP can regulate CXCR4 expression via knockdown of AFP in PLC/PRF/5 cells or appearance of AFP in pcDNA3.1-*afp* vectors transfected HLE cells. The data from both studies suggested a functional correlation between cytoplasmic AFP and the expressions of p-AKT(Ser473), p-mTOR(Ser2448), and CXCR4 as well as an interaction between AFP and PTEN, and our previous study[10] demonstrated that AFP binding with PTEN rendered it inactive and led to the concomitant increase expression of in p-AKT(Ser473) expression. Furthermore, pretreatment with Ly294002 also prevented AFP-induced mTOR phosphorylation and CXCR4 expression, confirming that mTOR activation and CXCR4 expression are dependent on AKT activity.

Although document it has been reported that the CXCR4 promoter region contains some potential transcription factor binding sites such as an NF-κB binding site[13], the promoter regulatory elements involved in *CXCR4* gene activation have not yet been verified. Interestingly, in this study, ChIP with p-mTOR(Ser2448) as a target showed a physical interaction between p-mTOR(Ser2448) and the *CXCR4* gene promoter when HLE cells were transfected with an AFP-expressing vectors that could be abolished by the silencing of AFP in PLC/PRF/5 cells, thereby providing the direct evidence that cytoplasmic AFP acts as a key regulator in the modulation of CXCR4 expression via activating the AKT/mTOR signaling pathway. Some studies have shown that CXCR4 plays a pivotal role in on metastasis of cancer cell[33,34], while we found that AFP stimulated CXCR4 expression by activating PI3K/AKT signaling[31]. In this study, the results also indicated that AFP harbors a function to stimulate migration of HCC, this investigation demonstrated that AFP promoted expression of CXCR4 contributes to metastasis of HCC cells.

To date, several studies[10,35-37] have focused on the biological role of cytoplasmic AFP as an important signaling molecule in the regulation of cell growth and apoptosis rather than just a well-defined diagnostic biomarker for HCC. However, thus far, few studies have attempted to identify the linkage of intracellular AFP with tumor invasion and metastasis, and the intrinsic mechanisms involving the correlation between AFP and CXCR4 have never been investigated. Recently, we had found that HBV-X protein priors to stimulate AFP expressed to promote expression of CXCR4 in HCC cells[38]. The findings of the present study indicated that AFP harbors a function to activate transduction of PI3K/AKT signal pathway via inhibiting PTEN activity, which contributes to HCC cells migration *in vitro*. The result from our present study adds to what is known about the physiological role of AFP; Combined with the results of previous studies, it is intriguing to realize that AFP has multiple complex regulatory roles in the promotion of liver cancer development and progression, this implicated that AFP was a novel target for the therapeutics of HCC.

In conclusion, AFP harbors a function to activate PI3K/AKT signal pathway to promote expression of CXCR4 in hepatoma cells, AFP stimulated migration of HCC cells through promoting expression of CXCR4. AFP was applied as a bio-target for therapeutic of HCC patients.

## MATERIALS AND METHODS

### Patients and specimens

The archived clinical specimens were originally collected during hepatectomy of 65 patients at Hainan Provincial People's Hospital (Haikou, Hainan, China) and the Affiliated Hospital of the Hainan Medical College (Haikou, Hainan, China) between January 2009 and September 2013. Of the 65 patients, 45 were men and 20 were women with a mean age of 49.6 (range, 22-76) years. All enrolled patients were treated with radical surgery and received no other treatments. Hepatitis B virus (HBV) infection was diagnosed by a serum hepatitis B surface antigen test, while circulating AFP plasma level was measured by enzyme-linked immunosorbent assay. Clinical data were obtained by a retrospective chart review. Follow-up was available for all patients. A section of liver tissue about 2.0×2.0×2.0 cm was obtained from each patient immediately after the surgery. About 1.0×1.0×1.0 cm tissue samples were fixed in 10% formalin, embedded in paraffin, and routinely stained with hematoxylin and eosin. The specimens were assessed blindly and independently by two pathologists. In case of interobserver disagreement, final decisions were achieved by general consensus. All selected patients were diagnosed by histopathological evaluation. The 1.0×1.0×1.0 cm tissue specimens were stored in liquid nitrogen. The study protocol was approved by the Ethical Committee of Hainan Provincial People's Hospital and the Science Investigation Ethical Committee of Hainan Medical College. Written informed consent was obtained from all participants.

### Immunohistochemical analysis

The expression and cellular distribution of AFP and CXCR4 protein were assessed by immunohistochemical analysis. Five-millimeter-thick paraffin sections were deparaffinized and re-hydrated according to standard protocols, and heat-induced antigen retrieval was performed in sodium citrate buffer (10mmol/L, pH 6.0). Endogenous peroxidase was inhibited by 0.3% H_2_O_2_, and non-specific protein binding was blocked with 10% goat serum. The sections were then incubated with primary antibody against AFP and CXCR4 (1:100 dilution; Santa Cruz Biotechnology Inc., Santa Cruz, CA, USA) at 4°C overnight. Non-immune immunoglobulin G(IgG) was used as a negative control, and antigenic sites were localized using a SP9000 Polymer Detection System and a 3,3′-diaminobenzidine kit (ZSGB-BIO, Beijing, China).

### Cell culture

The AFP-producing cell line PLC/PRF/5 and the non-AFP-producing cell line HLE[20] were gifts from the Department of Cell Biology, Peking University Health Science Center(Beijing, China) and were grown in Dulbecco's modified Eagle's medium (Gibco, Carlsbad, CA, USA) supplemented with 10% fetal calf serum(FCS) (Gibco) and 100 U/mL penicillin and 100 μg/mL streptomycin. All cell lines were cultured at 37°C in a humidified atmosphere with 5% CO_2_.

### RNA interference

For the RNA interference(RNAi) experiments, the anti-AFP-specific siRNA-expressing vectors (AFP-siRNA923) directed at the 923-944 region of the *AFP* gene and a corresponding scrambled sequence as the negative control were used in this study as described previously[9,10]. After the transfection was induced by Lipofectamine 2000(Invitrogen, Carlsbad, CA, USA), the AFP-producing PLC/PRF/5 cells were incubated with AFP-siRNA for 6 h and the medium was replaced with complete medium for 42 h. The cells were then harvested for western blotting, co-immunoprecipitation(Co-IP) and chromatin immunoprecipitation(ChIP) assay. These tests were performed as described previously[9,10].

### Generation of an AFP-expressing construct

The AFP-expressing construct(pcDNA3.1-*afp*) was created as described previously[9]. The transient transfections were conducted in non-AFP-producing HLE cells (1×10^5^ cells/well in a 12-well plate for a confluent cell layer) using Lipofectamine 2000(Invitrogen) in Opti-MEM reduced serum medium(Invitrogen).

### Immunofluorescent staining

Indirect immunofluorescence was performed on HCC cells cultured on glass coverslips. After overnight incubation with primary antibodies against AFP, CXCR4, and PTEN (1:100, Santa Cruz Biotechnology) at 4°C, the antigenic sites were detected using fluorescein-5 isothiocyanate-conjugated(FITC) goat anti-mouse or tetramethylrhodamine isothiocyanate-conjugated(TRITC) goat anti-rabbit IgG (1:100; Protein Tech Group, Inc., Chicago, IL, USA), and the cells were incubated with DAPI (1 μg/mL) for 30 minutes. Images of the antigenic sites were captured with a laser scanning confocal microscope (TCS SP5 II; Leica, Solms, Germany).

### Protein expression evidenced by Western blotting

Total proteins were extracted using RIPA lysis buffer (Beyotime Institute of Biotechnology, Jiangsu, China). The proteins (50 μg total) were subjected to sodium dodecyl sulfate-polyacrylamide gel electrophoresis and transferred to polyvinylidene fluoride membranes. After incubating with 5% skim milk in Tris-buffered saline and Tween-20(TBST) at 37°C for 30 min, the membranes were probed for the following primary antibodies: mouse anti-AFP (1:500), -AKT or mTOR (1:500) or -β-actin (1:1000); rabbit ant-CXCR4 (1:400), -p-AKT(Ser473) (1:500), or -p-mTOR(Ser2448) (1:500) antibody(all from Santa Cruz Biotechnology Inc.) overnight at 4°C. After three washes with TBST, the membranes were incubated with horseradish peroxidase-conjugated secondary antibodies for 1 hour at 37°C. The bands were visualized using enhanced chemiluminescence reagents (Thermo Fisher, Rockford, IL, USA) and analyzed with a gel analysis system (VersDoc TM5000MP System; BIO-RAD, Guangzhou, China). The expression of β-actin was used as loading control.

### Co-immunoprecipitation(Co-IP) Assay

A Co-IP kit (Pierce Corp, Rockford, IL, USA) was used to evaluate the interaction between AFP and PTEN in HCC specimens and PLC/PRF/5 cells and HLE cells as described previously[19]. Antibodies were purchased from Santa Cruz Biotechnology.

### Chromatin immunoprecipitation(ChIP) Assay

To define the interaction of p-mTOR(Ser2448) with the CXCR4 regulatory motif, a ChIP assay kit (Epigentek Corp, Farmingdale, NY, USA) was used as described previously[39]. The sequence for the human CXCR4 gene promoter was as follows: forward, 5′-GGCAGCAGGTAGCAAAGTGA-3′; reverse, 5′-AGACAATGTAACT CGCTCCAAGA-3′. The PCR products were analyzed on 2% agarose gel electrophoresis and documented.

### Wound healing assay

Cells motility was analyzed by a wound healing assay. One day before scratching, HLE cells transfected with pcDNA3.1-*afp* and PLC/PRF/5 cells transfected with AFP-siRNA were seeded into 12-well plates to almost total confluence in 24 hours. A scratching wound was made by scraping the middle of the cell monolayer with a sterile micropipette tip. After all detached cells were washed away with PBS, the cells were cultured with medium containing 10% FCS, images of the cells migrating into the wound were captured at 0 and 24 hours by an inverted microscope (100×), and their distances were recorded. Cell-repaired motility was evaluated using the following formula: Cell repair ratio(%)=(distance 0 hour-distance 24 hours)/distance 0 hour ×100%.

### Transwell assay

To measure cell migration, culture inserts (Transwell; 8-mm pore size; Costar, High Wycombe, UK) were placed into the wells of 12-well culture plates and the upper and lower chambers were separated. Cells (5×10^4^) were added to the upper chamber and cultured with serum-free RPMI-1640 medium, whereas the lower chamber was filled with complete medium containing 50ng/mL stromal-derived growth factor-1. After 24 hours of incubation, the cells in the upper chamber were carefully removed with a cotton swab and those that had migrated through the membrane to the lower surface were fixed with 90% methanol and stained with 0.1% crystal violet. The number of cells that had migrated through the pores was quantified by counting five independent visual fields under the microscope(Olympus) using a 20×objective. Three independent assays were performed.

### Statistical analysis

The results of multiple observations are presented as mean±sd of at least three independent experiments. Statistical significance was determined using Student's t-test (SPSS 11.5 software for Windows, SPSS Inc., Chicago, IL, US).

## Abbreviations

AFP: alpha-fetoprotein
Co-IP: co-immunoprecipitation
ChIP: Chromatin immunoprecipitation
DMEM: Dulbecco's Modified Eagle Medium
FCS: fetal calf serum
PI3K: phosphatidylinositol 3-kinase
AKT: protein kinase B
pAKT: phosphorylated AKT(Ser473)
mTOR: Mammalian target of rapamycin
p-mTOR: phosphorylated mTOR(Ser2448)
PTEN: phosphatase and tensin homolog deleted on chromosome 10
CXCR4: CXC motif chemokine receptor 4
TRITC: tetramethylrhodamine isothiocyanate
FITC: Fluorescein-5-isothiocyanate
